# The wild mouse bone marrow has a unique myeloid and lymphoid composition and phenotype

**DOI:** 10.1093/discim/kyad005

**Published:** 2023-04-18

**Authors:** Andrew Muir, Alex Bennett, Hannah Smith, Larisa Logunova, Andrew Wolfenden, Jonathan Fenn, Ann E Lowe, Andy Brass, John R Grainger, Joanne E Konkel, Janette E Bradley, Iris Mair, Kathryn J Else

**Affiliations:** Lydia Becker Institute of Immunology and Inflammation, School of Biological Sciences, Faculty of Biology, Medicine and Health, University of Manchester, Manchester, UK; Lydia Becker Institute of Immunology and Inflammation, School of Biological Sciences, Faculty of Biology, Medicine and Health, University of Manchester, Manchester, UK; Lydia Becker Institute of Immunology and Inflammation, School of Biological Sciences, Faculty of Biology, Medicine and Health, University of Manchester, Manchester, UK; Lydia Becker Institute of Immunology and Inflammation, School of Biological Sciences, Faculty of Biology, Medicine and Health, University of Manchester, Manchester, UK; School of Life Sciences, University of Nottingham, Nottingham, UK; School of Life Sciences, University of Nottingham, Nottingham, UK; School of Life Sciences, University of Nottingham, Nottingham, UK; School of Health Sciences, The University of Manchester, Manchester, UK; Lydia Becker Institute of Immunology and Inflammation, School of Biological Sciences, Faculty of Biology, Medicine and Health, University of Manchester, Manchester, UK; Lydia Becker Institute of Immunology and Inflammation, School of Biological Sciences, Faculty of Biology, Medicine and Health, University of Manchester, Manchester, UK; School of Life Sciences, University of Nottingham, Nottingham, UK; Lydia Becker Institute of Immunology and Inflammation, School of Biological Sciences, Faculty of Biology, Medicine and Health, University of Manchester, Manchester, UK; Lydia Becker Institute of Immunology and Inflammation, School of Biological Sciences, Faculty of Biology, Medicine and Health, University of Manchester, Manchester, UK

**Keywords:** wild mouse, bone marrow, leukocytes, ecoimmunology, flow cytometry, hematopoietic progenitors

## Abstract

The murine bone marrow has a central role in immune function and health as the primary source of leukocytes in adult mice. Laboratory mice provide a human-homologous, genetically manipulable and reproducible model that has enabled an immeasurable volume of high-quality immunological research. However, recent research has questioned the translatability of laboratory mouse research into humans and proposed that the exposure of mice to their wild and natural environment may hold the key to further immunological breakthroughs. To date, there have been no studies providing an in-depth cellular analysis of the wild mouse bone marrow. This study utilized wild mice from an isolated island population (Isle of May, Scotland, UK) and performed flow cytometric and histological analysis to characterize the myeloid, lymphoid, hematopoietic progenitor, and adipocyte compartments within the wild mouse bone marrow. We find that, compared to laboratory mouse bone marrow, the wild mouse bone marrow differs in every cell type assessed. Some of the major distinctions include; a smaller B cell compartment with an enriched presence of plasma cells, increased proportions of KLRG1+ CD8+ T cells, diminished CD11b expression in the myeloid lineage and a five-fold enlargement of the eosinophil compartment. We conclude that the wild mouse bone marrow is dramatically distinct from its laboratory counterparts, with multiple phenotypes that to our knowledge have never been observed in laboratory models. Further research into these unique features may uncover novel immunological mechanisms and grant a greater understanding of the role of the immune system in a natural setting.

## Graphical Abstract

**Graphical Abstract:**
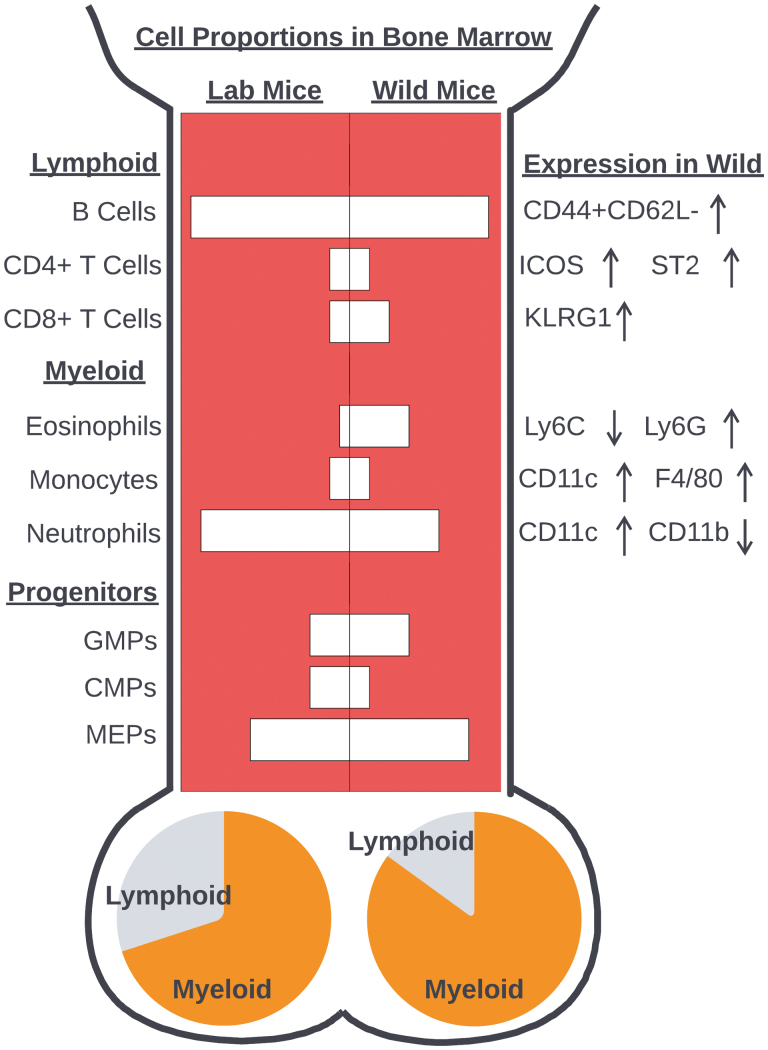

## Introduction

*Mus musculus domesticus*, commonly known as the house mouse, is the most well-studied and commonly used model species in immunology, owing to their ease of husbandry and a mammalian immune system comparable to humans [1]. However, the requirements to rear mice in laboratory conditions limits the potential for research into immune–environmental interactions. Wild mice present an opportunity to study how the environment impacts on immune function in a real-world setting. Wild mice are immunologically distinct from laboratory mice [2], and in some aspects, the phenotype of wild mice mirrors the human immune phenotype more closely than laboratory mice do [3, 4]. The Isle of May (Scotland, UK) has precedence in the field of mouse research with the works by G.S. Triggs and R.J. Berry providing important background knowledge [5–8]. As an isolated island habitat with extremely limited human interactions, the Isle of May is ideal for studying the impacts of natural environmental variables on immunological, ecological, and health based metrics.

As the single largest source of leukocytes in adult mice [9], the bone marrow is a crucial component of the immune system. In addition to the production of leukocytes, the bone marrow is also a major modulator of immune function, as transcriptional changes in progenitor populations alter the phenotype of progenitor-derived leukocytes [10, 11]. Environment linked factors such as infection, diet, and the microbiota are well-studied modulators of bone marrow function [12, 13], driving systemic changes in immune responses. Given the bone marrow’s central and systemic influence on the immune system, we hope to further our understanding of immune–environment interactions by better characterizing the impact of the natural environment on bone marrow phenotype.

As there have been no prior studies providing an in-depth cellular analysis of the wild mouse bone marrow, this study characterizes the composition and phenotype of the wild mouse bone marrow and provides a contrast with its laboratory counterpart. Specifically, this study assesses the composition and phenotype of B cells, CD4+ T cells, CD8+ T cells, eosinophils, neutrophils, monocytes, leukocyte progenitors, and adipocytes to provide a broad view of the composition of the wild mouse bone marrow. To provide contrast, C57BL/6 mice (the most commonly used laboratory strain) and ICR mice (a common outbred strain derived from CD-1) were chosen, to be used within a long-term life-cycle stress model using 6-month old, retired female breeders that had been utilized as breeding stock since maturity. Pregnancy and ageing are combinatorial factors that alter haematopoiesis over the murine life cycle [14]. Using these models, we have identified multiple novel phenotypes unique to the wild mouse bone marrow. These include enrichment in plasma cells, increased proportions of KLRG1+ CD8+ T cells, diminished CD11b expression in the myeloid lineage and a 5-fold enlargement of the eosinophil population. The results of this study are a foundation and an exciting preview to incentivize further wild mouse immunology research.

## Materials and methods

### Mice

For laboratory mice, 10 mice each of C57BL/6 and ICR (derived from CD-1^®^) were utilized (CD-1^®^ is a registered trademark of Charles River Laboratories, Massachusetts, USA). Five mice of the C57BL/6 strain were provided ‘in-house’ by the Biological Services Facility at the University of Manchester, these mice were all females and aged 5 weeks. Five C57BL/6 and 10 ICR mice were purchased from ENVIGO (Blackthorn, UK). All mice were female, five ICR mice were 5 weeks of age, while five C57BL/6 and five ICR were 6 months of age and classed as retired breeders (previously utilized as stock breeders). Laboratory mice were used under license P47AFBF29. All wild mice were used under project licence P6427E971. All mice were euthanized with rising concentrations of CO_2_. After euthanasia, both femurs were dissected, and any remaining attached tissue removed. One femur was used for histological analyses and one for flow cytometric analyses as detailed below.

All work involving wild mice was performed on the Isle of May (56°11ʹ11.6ʹʹN, 2°33ʹ24.1ʹʹW), located off the south-east coast of Scotland in the Firth of Forth. Wild house mice (*M. m. domesticus*) were live-trapped across three sessions from November 2019 to November 2021, with each session including four trapping days. Two trapping grids of 96 Longworth traps, each placed 8–10 m apart in a 6 × 16 grid, were setup in two separate regions. Traps were baited with sunflower seeds, lined with bedding and were opened each evening. Less than 9 h later, triggered traps were collected and any non-triggered traps closed for the duration of the day. Mice were either culled at first capture or released and culled at later recapture. Mice released for recapture were subcutaneously injected with a Passive Integrated Transponder tag for identification upon later recapture.

Typical mouse demographical metrics and prevalence of parasite infections are summarized in Supplementary Table 4. Demographics of bone marrow sample collection are illustrated in Supplementary Fig. 1.

### Preparation of bone marrow for histology

Whole femurs were submerged in 10% NBF for 24 h at room temperature then stored in 70% ethanol at room temperature until further processing. To decalcify, femurs were submerged in decalcification solution (PBS pH7.2, 15% EDTA, 1% NBF) for 2 weeks at room temperature. Femurs were then dehydrated in 70% ethanol prior to embedding in paraffin. Paraffin-embedded femurs were sectioned at 8 µm thickness on a Leica microtome. Sections were cut perpendicular to the length of the femur, through the central portion (not proximal nor distal).

### Bone marrow histology staining

Slides were deparaffinized by submersion in xylene for 20 min, then rehydrated through decreasing concentrations of ethanol (70%, 50%, 30% ethanol for 3 min each), then 3 min in distilled water. Slides were stained for 1 h with Giemsa (Sigma 48900) made up 1:200 in pH6.8 water. Post-staining, slides were rinsed for 10 s in pH6.8 water, then air dried. Slides were then mounted onto frosted non-adhesion slides using DPX non-aqueous mounting medium (Sigma 06522) and allowed to dry prior to imaging.

### Histology data processing

Giemsa-stained bone marrow slides were imaged using a Leica slide-scanner at 40× magnification to produce digital images. Digital images were viewed using CaseViewer (v2.4 3DHISTECH). Representative images were collected by taking sequential (non-overlapping) 40× magnification snapshots as a linear cross-section of each bone marrow sample. Sequential snapshots were collected until a full cross-section was recorded, varying between two and five images depending on the size of the femur section (median of three images per sample). Images were recorded at 3840 × 2066 resolution then downscaled to 1920 × 1033 resolution to accelerate later processing methods. The full data set included 151 samples and 404 images. Major bone marrow features (bone, marrow, and adipocytes) were automatically quantified using Trainable Weka Segmentation (plugin within the Fiji ImageJ distribution). Further details of this processing are included in the extended methods.

### Bone marrow extraction

The femur not used for histological analysis was placed in ice-cold PBS until ready to process for marrow extraction, with all marrow being harvested on the same day as mouse cull (less than 12 hours between cull and flow cytometry staining). To extract the bone marrow, each femur was rinsed for 1 min in 70% ethanol to sterilize, then rinsed in sterile PBS. Both ends of each femur were removed and marrow displaced by pressurizing sterile PBS through the bone cavity using a 5 ml syringe and 19 g needle. The marrow was then collected, centrifuged, and resuspended ready for flow cytometry staining.

### Bone marrow flow cytometry staining

Suspensions of bone marrow in PBS at 1 × 10^6^ cells/ml were stained with Fixable Viability dye eFluor 455UV (Thermo Fisher) and anti-CD16/CD32 (Thermo Fisher) in the dark for 10 min at 4°C (progenitor panel exclusively did not receive anti-CD16/32). Fluorochrome-conjugated antibodies in FACS buffer were then added and supplemented with Super Bright staining buffer (Thermo Fisher) for 30 min. Cells were washed, fixed in IC fixation buffer (Thermo Fisher) for 15 min at 4°C, washed again and re-suspended in FACS buffer.

The lymphoid panel additionally utilized intra-cellular staining, whereby cells were resuspended in fix-perm buffer (eBioscience #00-5223-56, eBioscience #00-5123-43) overnight (maximum 18 h) at 4°C. Next cells were washed in perm wash (eBioscience #00-8333-56) then stained with fluorochrome-conjugated antibodies in perm wash for 30 min at room temperature. Cells were then washed and resuspended in FACS buffer.

Post staining, samples were kept in the dark at 4°C and acquired within 3–7 days of preparation on an LSR Fortessa running FACSDIVA 8 software (BD). Acquisition parameters on FACSDIVA were kept identical throughout the study. FMOs (Flourescence Minus One) controls were prepared alongside samples using pooled cells from all mice culled on the day of staining. Staining procedures for FMOs were identical to samples except that FMOs had one fluorophore omitted for each FMO prepared.

For antibodies, concentrations and gating strategies used, see Supplementary Figs 2–4. All fluorochrome-conjugated antibodies were purchased from Life Technologies (Paisley, UK).

### Statistical analyses

All calculations of *P* values were performed using a Kruskal–Wallis test followed by a post-hoc Dunn’s test with Bonferroni correction, except for Fig. 5F and G which use the Mann–Whittney *U* test. A full table of all *P*-values calculated by Kruskal–Wallis and Dunn’s tests is provided as Supplementary Table 2.

## Results and Discussion

### Increased leukocytes with a myeloid bias and a distinct arrangement of leukocyte progenitors indicate a unique developmental environment for wild mouse bone marrow leukocytes

**Figure 1:**
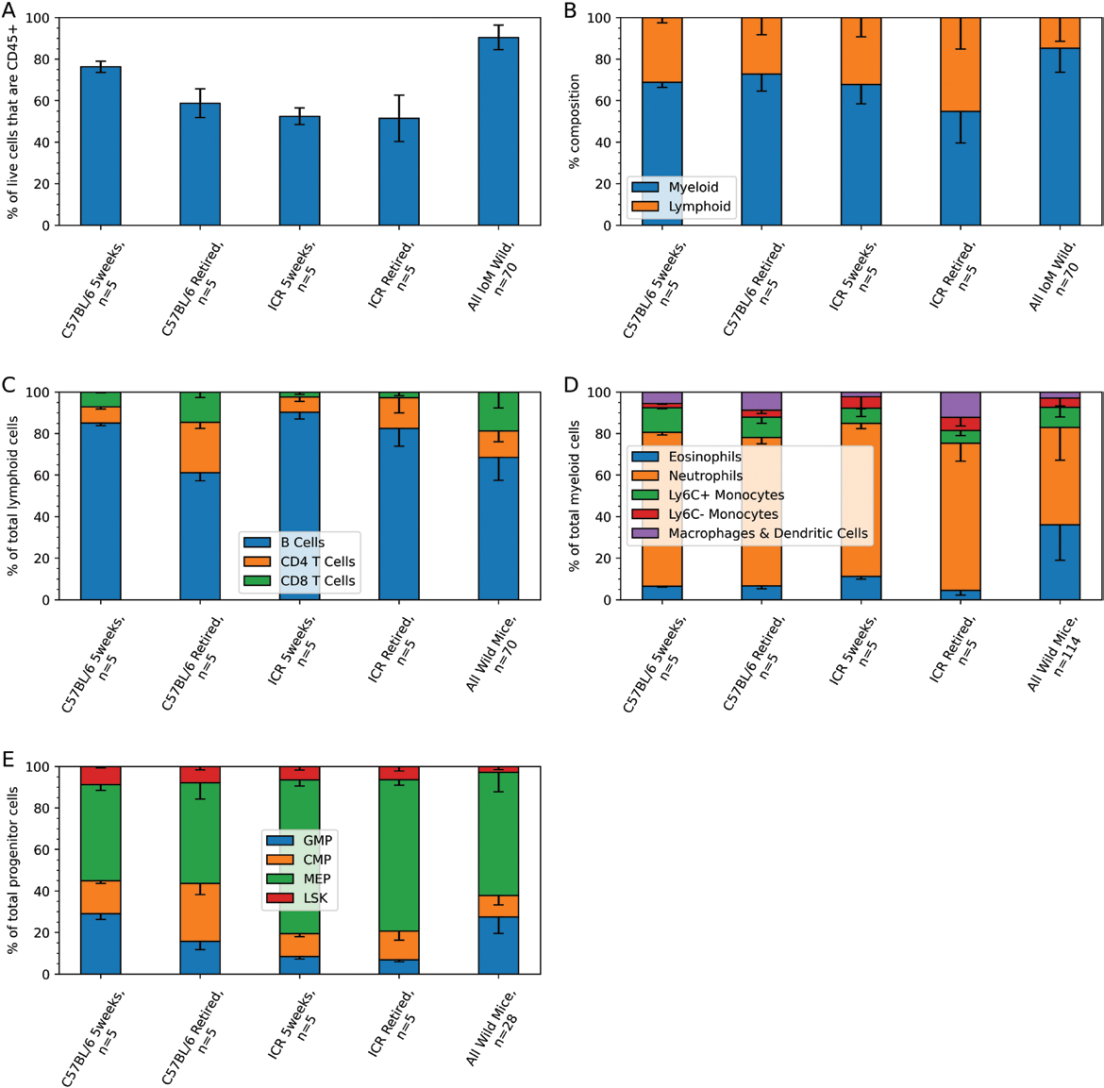
Leukocyte and progenitor composition of the laboratory and wild mouse bone marrow. Bone marrow extracted from mouse femurs was stained with fluorescent antibodies (Supplementary Figs 2–4) and cell types identified using flow cytometry (gating strategies in Supplementary Figs 2–4) with the identifying markers used summarized in Supplementary Table 1. (A–E) ‘5weeks’ are 5-week-old female mice, ‘retired mice’ are 6-month-old females with a history as stock breeders. ‘All Wild Mice’ includes all Isle of May wild mice for which data is available. Bar charts show the mean percentage of each cell population, error bars show one standard deviation from the mean, (B–E) positive error bars have been omitted to improve legibility. *P*-values for each set of comparisons (each cell type) were calculated by performing a Kruskal–Wallis test followed by a post-hoc Dunn’s test with Bonferroni correction and are included in Supplementary Table 2 (not displayed in figure). (D) Error bars for the combined macrophage and dendritic cell proportions have been omitted to improve legibility.

CD45 is primarily expressed by leukocytes (and some progenitors and stromal cells) [15] and can be used as a crude estimate of the overall leukocyte composition in the bone marrow. Wild mice showed a substantial increase in CD45+ proportion (out of all live cells) versus all laboratory mouse groups (Fig. 1A, *P* < 0.01).

It is well established that ageing leads to an increasing myelopoeisis bias in both mice and humans [16, 17] and life cycle stresses such as pregnancy can both exasperate and mitigate these effects [14]. In the mouse, the main myeloid progenitors are common myeloid progenitors (CMP), which differentiate from multipotent stem cells (MPP) and give rise to megakaryocyte erythrocyte progenitors (MEP) and granulocyte monocyte progenitors (GMP) [18]. However, comparing 5 week and retired laboratory mice (Fig. 1B and E), there was no significant increase in the myeloid/lymphoid ratio though there was a decrease in the GMPs (*P* = 0.038). The absence of any changes in the myeloid/lymphoid ratio in the current study may be explained by the fact that the retired breeders were only 6 months old, with the age asscociated increases in myelopoieisis typically only arising in mice at a much older 1–2 years of age [16, 17]. Further, repeated pregnancies may have slowed the ageing process [14]. When comparing the myeloid/lymphoid ratios between laboratory mice and wild mice (Fig. 1B), there was a myeloid bias in wild mice (*P* < 0.02). Comparing C57BL/6 5 weeks versus wild mice (Fig. 1E), wild mice had a halved LSK proportion (*P* < 0.01), a reduced CMP proportion (*P* < 0.01), increased MEPs (*P* = 0.015) but similar GMPs (*P* = 0.70). In contrast, GMPs were increased in wild mice when compared with either of the ICR groups (*P* < 0.01), but MEPs were reduced (*P* < 0.025) and CMPs were similar (*P* > 0.12). Overall, the wild mouse bone marrow had a myeloid bias and an increase in the CD45+ proportion, though whether this stems from increased myelopoieisis (particularly GMPs or CMPs) is not clear from this data alone. Ideally, this myeloid bias would be contrasted with the ages of the wild mice; however, we currently only have approximate estimates of mouse age via dry eye lens weight and a multi-parameter proxy including dry eye lens weight, body weight, body length, and tail length. These two estimation methods of mouse age were compared against myeloid bias in the wild mice but neither returned significant correlation (data not shown). Beyond ageing, both acute and chronic infections are known to stimulate myelopoieisis [13], both *Trichuris muris* and *Calodium hepaticum* are endemic to the Isle of May mouse population, and given the natural environment it is expected that the wild mice are constantly exposed to a wide range of other pathogens.

### The wild mouse bone marrow has a small, but effector enriched B cell compartment

**Figure 2:**
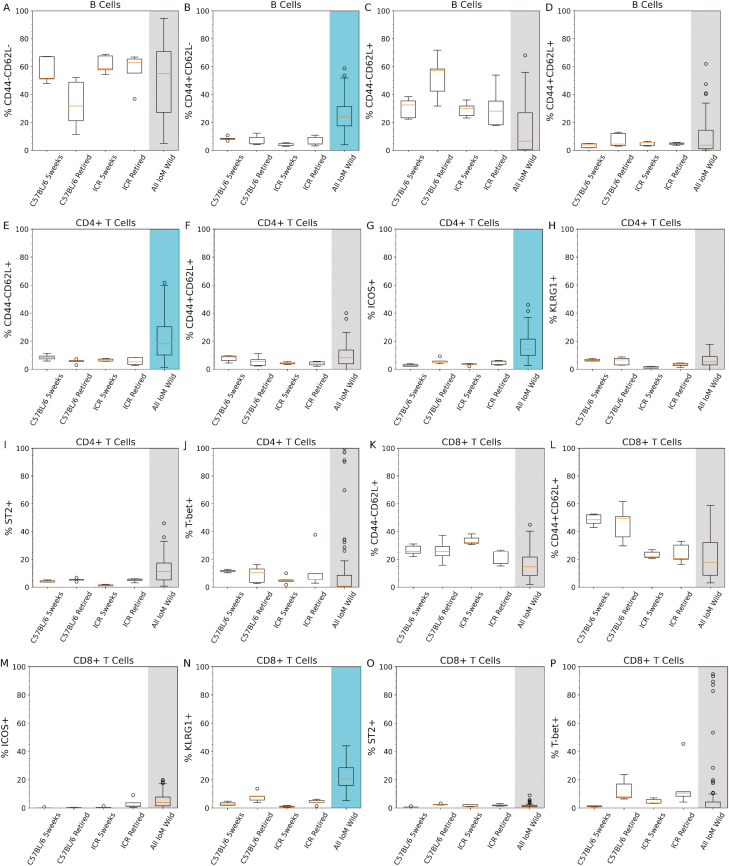
Lymphoid cell phenotypes in laboratory and wild mouse bone marrow. Bone marrow extracted from mouse femurs was stained with fluorescent antibodies from panel A (Supplementary Fig. 2) and cell types identified using the lymphoid gating strategy (Supplementary Fig. 2). Y-axis shows the % cell expression of the indicated marker, the positions of gates for cell marker expression is provided in Supplementary Fig. 2. ‘5-weeks’ are 5-week-old naive female mice, ‘retired mice’ are 6-month-old females with a history as stock breeders. ‘All IoM Mice’ includes all Isle of May wild mice for which data is available. Box plots follow Tukey’s original definition: The orange line is the median, box limits show the interquartile range (IQR), whiskers are set to the closest value below 1.5 times the IQR, outliers (identified by being more than 1.5 times the IQR) are displayed as round circles. p-values for each set of comparisons (each individual plot) were calculated by performing a Kruskal–Wallis test followed by a post-hoc Dunn’s test with Bonferroni correction and are included in Supplementary Table 2 (not displayed in figure). When the Dunn’s test *P*-values between the wild mice group and all of the laboratory mouse groupings was lower than 0.05 the wild mice group has been shaded blue, where at least one of the *P*-values were above 0.05 the wild mouse group has been shaded grey. *n* = 5 for each laboratory mouse group and *n* = 70 for the all wild mice group (all wild mice for which lymphoid data is available).

Previous studies have shown that the mouse bone marrow is enriched with long-lived plasma cells compared to other lymphoid organs [19] and is an important site for lymphocyte sequestration during stress [20]. In our study, compared to a 5-week-old C57BL/6 mouse, the wild mouse bone marrow, had a 20% smaller proportion of B cells (out of all lymphoid cells, Fig. 1C) with a greatly expanded proportion of CD44+CD62L- effector B cells (median of 55% versus 5%, Fig. 2B) and smaller CD44-CD62L+ naive B cell proportion (median of 7% versus more than 26%, Fig. 2C). Versus all the laboratory mouse groups, the CD44+CD62L- cohort of B cells was significantly higher in the wild mouse population (*P* < 0.01). However, it cannot be ascertained with this data if this skew is due to alterations in B cell development (driving increased mature B cells that indirectly increases effectors), increased B cell activation (directly increasing B cell effectors), or increased sequestration of B cells from other lymphoid organs (increasing the proportion of effectors).

### An increased proportion of naive and ICOS+ CD4+ T cells differentiates the wild mouse bone marrow CD4+ T cell compartment from all laboratory equivalents

The proportion of CD4+ T cells within the bone marrow lymphoid population of wild mice was higher than 5-week-old laboratory mice, but lower than retired laboratory mice (both C57BL/6 and ICR) (Fig. 1C). This was despite the wild mouse bone marrow showing a wide range of CD4+ T cell proportions, ranging from 9% to 25% of all lymphoid cells. This presents a strong argument that the CD4+ T cell proportion (out of lymphoid cells) increases with age, as has been noted elsewhere [16, 17] and would place the age of the captured wild mice within a range between the 5-week-old and 6-month-old mice, which is consistent with the survey data provided by Triggs [8] on the estimated age ranges of captured Isle of May mice.

Comparing laboratory versus wild mice, CD44-CD62L+ and ICOS+ consistently discriminated between the laboratory and wild mice groups (Fig. 2E–J). Wild mice had a larger CD44-CD62L+ CD4+ T cell proportion (*P* < 0.042) and higher ICOS+ proportion (*P* < 0.01) when compared with laboratory mouse CD4+ T cells. ST2 expression in wild mouse bone marrow CD4+ T cells was significantly increased versus both 5-week-old laboratory mouse groups (*P* < 0.015) but was not significant when compared against either of the 6-month-old mouse groups (*P* < 0.11). Additionally, the wild mice showed greatly increased variability in all measured CD4+ T cell biomarkers when compared with any of the laboratory mice groups. Intriguingly, there was not a significant difference in either effector (CD44+CD62L-, *P* > 0.07) or memory (CD44+CD62L+, *P* > 0.07) CD4+ T cell proportions versus laboratory mouse bone marrow (data not shown), contrary to findings in the literature investigating the phenotype of splenic CD4+ T cells in wild mice [2, 3].

ICOS is a costimulatory receptor that is rapidly upregulated upon T cell activation, is utilized for reactivation of memory cells and is required for T helper cells to assist in B cell class-switching [21, 22]. Similarly, ST2 is the receptor for IL-33 and is heavily involved in Th2 activation and type 2 responses [23]. The increase in ICOS+ and ST2+ proportions across the wild mouse population but lack of a statistically significant difference in either effector CD4+ T cell proportions versus laboratory mouse bone marrow could indicate an immunologically primed state and a greater priority for assistance for B cell activation, which aligns with the higher average proportions of B cell effectors observed in wild mice.

### The senescence marker KLRG1 is highly expressed in wild bone marrow CD8+ T cells

The bone marrow CD8+ T cell compartment in the wild and laboratory mouse can be differentiated by a higher proportion of KLGR1+ CD8+ T cells in the wild mice (Fig. 2K–P, *P* < 0.01 versus all laboratory groups). For all other CD8+ T cell markers, while there was no substantial difference in average values between laboratory and wild mice, there was a considerable increase in variability of expression in the wild mice. KLRG1 in CD8+ T cells is typically seen as a negative regulator of activation expressed by chronically stimulated or senescent CD8+ T cells [24]. KLRG1 upregulation by CD8+ T cells has been found to be driven by persistent antigen stimulation and chronic viral infections [25, 26]. Given the wild environment of the mice, it is viable to postulate that the increased proportion of KLRG1+ CD8+ T cells in the bone marrow may be due to chronic antigenic exposure.

### The wild mouse bone marrow has a uniquely large and phenotypically distinct eosinophil compartment

In the laboratory mouse literature, bone marrow eosinophils are a minor population constituting only 2.5% of the murine bone marrow’s hematopoietic composition [27]. Accordingly, eosinophils comprised less than 10% of the mouse bone marrow myeloid compartment for all laboratory mouse groups (Fig. 1D). However, in the wild mouse, the eosinophil composition ranged from 10% up to 65% (of all myeloid cells) and was significantly higher (*P* < 0.01) than all of the laboratory mouse groups. This dramatic reorganization of the murine bone marrow myeloid compartment is unprecedented; homologues of bone marrow eosinophilia can be found in allergic airway inflammation, myelodysplastic syndrome, drug-induced hypersensitivity, and tissue invasive worm infections, albeit in humans more often than mice [28]. However, these studies all report an eosinophilic composition of less than 10% of the hematopoietic bone marrow compartment. Of the above mentioned diseases only tissue-invasive worms are relevant to the Isle of May mice. Indeed, the IoM mice are known to be hosts for *Calodium hepaticum*, a liver dwelling parasite. However, a separate investigation (data not included) found only a very weak correlation between *Calodium* burden and eosinophil phenotype. Similarly, *Trichuris muris* infection is endemic within the Isle of May wild mouse population, but severity of *Trichuris muris* burden at cull has shown no significant correlation with bone marrow eosinophil phenotype (data not shown). Hence, it is likely there are other factors (such as other parasites or historical infections) that drive this eosinophil dominant phenotype.

**Figure 3:**
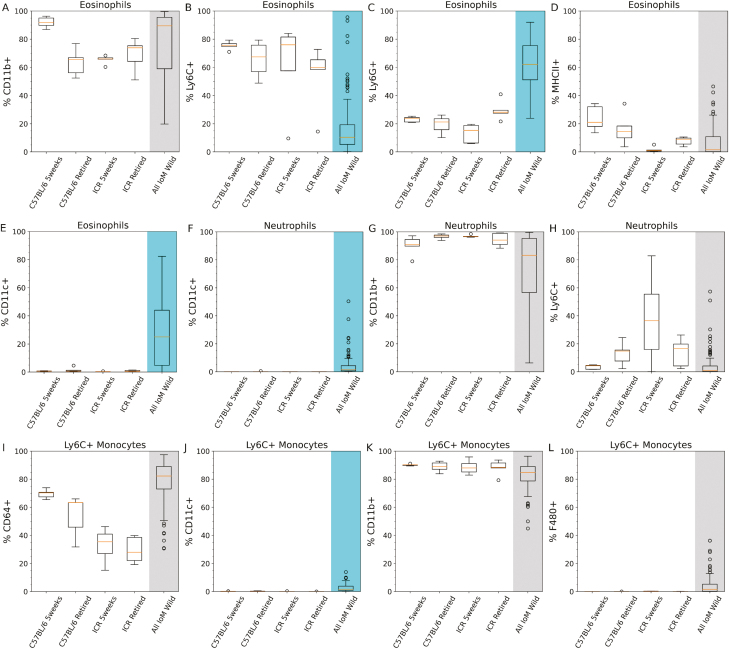
Myeloid cell phenotypes in laboratory and wild mouse bone marrow. Bone marrow extracted from mouse femurs was stained with fluorescent antibodies from panel B (Supplementary Fig. 3) and cell types identified using the myeloid gating strategy (Supplementary Fig. 3). Y-axis shows % cell expression of the indicated marker, the positions of gates for cell marker expression is provided in Supplementary Fig. 3. ‘5weeks’ are 5-week-old naive female mice, ‘retired mice’ are 6-month-old females with a history as stock breeders. ‘All IoM Mice’ includes all Isle of May wild mice for which data is available. Box plots follow Tukey’s original definition: The orange line is the median, box limits show the interquartile range (IQR), whiskers are set to the closest value below 1.5 times the IQR, outliers (identified by being more than 1.5 times the IQR) are displayed as round circles. *P*-values for each set of comparisons (each individual plot) were calculated by performing a Kruskal–Wallis test followed by a post-hoc Dunn’s test with Bonferroni correction and are included in Supplementary Table 2 (not displayed in figure). When the Dunn’s test *P*-values between the wild mice group and all of the laboratory mouse groupings was lower than 0.05 the wild mice group has been shaded blue, where at least one of the *P*-values were above 0.05 the wild mouse group has been shaded grey. *n* = 5 for each laboratory mouse group and *n* = 114 for the all wild mice group (all wild mice with myeloid data).

Laboratory mouse eosinophils are typically Ly6C^hi^ and Ly6G^lo^ [15, 29]. Comparing laboratory versus wild mice (Fig. 3A–E), the Ly6C+ proportion was lower in wild mice (*P* < 0.01) and the Ly6G+ proportion was higher in wild mice (*P* < 0.01). Overall, we can define wild mouse bone marrow eosinophils as Ly6C^var^ and Ly6G^hi^. This is consistent with our previous study [30] describing eosinophils in peritoneal exudate cells, spleen, and bone marrow of the Isle of May wild mice, though this new study greatly expands the sample population (114 mice versus 10 mice) and further identifies Ly6C and CD11c expression (see next paragraph) as distinct from laboratory counterparts. The functional role of the Ly6 proteins is far from clear, instead they are commonly used as surface markers for leukocyte identification [15, 31]. However, increased Ly6G expression by eosinophils has been observed during fungal allergen challenge [32] and can be induced via IL-5 administration [33]. Finding that wild mouse bone marrow eosinophils have a distinct Ly6C/Ly6G identification profile is a novel and important finding in itself and would be worth further morphological and functional assessment in future studies.

Laboratory mouse bone marrow eosinophils are typically CD11c- however this study found that wild mouse bone marrow have highly variable CD11c+ eosinophil proportions (Fig. 3E) that was significantly higher than any of the laboratory mouse groups (*P* < 0.02). A CD11c^hi^ phenotype in eosinophils is typically associated with a chronic inflammatory response [34, 35], hence the variation in CD11c+ proportion in the wild mouse eosinophil population may be due to the incidence of underlying inflammatory processes.

### Comparable proportions but a distinct phenotype for Ly6C+ monocytes in the wild mouse bone marrow

There were minimal difference in the proportions of monocytes between the laboratory and wild mouse groups (Fig. 1D). However, there was a clear division in biomarker expression, with CD11c (*P* < 0.01) being expressed by a greater proportion of bone marrow monocytes in wild mice versus all of the laboratory mice groups (Fig. 3I–L). However, the proportions of both CD64 and F4/80 expressing bone marrow monocytes were highly variable within the wild mouse population. CD64 on monocytes is rapidly upregulated during the initiation and progression of active infections [36, 37], hence this variable expression pattern may represent mice with different stages, types, or combinations of active infection.

### Low expression of CD11c and a variable CD11b expression characterizes a novel neutrophil phenotype

In the laboratory mouse bone marrow literature, neutrophils are the most abundant haematopoietic cell type, making up around 37% of all hematopoietic cells and 70% of all myeloid cells [27]. However, one of the most striking observations in the wild mouse bone marrow was the dramatic reduction in neutrophil proportions in the majority of the wild mice (Fig. 1D) and concurrent increase in eosinophil proportions. Potentially this dramatic shift in neutrophil to eosinophil proportions could be related to the recently documented potential for ‘mature’ murine bone marrow neutrophils to still have the capacity to differentiate into eosinophils or monocytes [38]. Specifically, Jeong *et al* [38]. characterized a SiglecF-, Ly6G+, IL-5Rα+ population, which would traditionally be considered to be neutrophils (despite IL-5Rα typically being associated with eosinophils), to have multipotent myeloid cell properties. Even more surprising is the observation that this population described by Jeong [38] was the dominant neutrophil population in the bone marrow and increased with age. As per Supplementary Fig. 3, the neutrophils described here are SiglecF- and Ly6G+, whereas eosinophils are SiglecF+ Ly6G^var^. Unfortunately, without the inclusion of IL-5Rα in the flow panel it is not possible to tell if the wild bone marrow neutrophil/eosinophils belong to the same population as described by Jeong *et al* [38]. However, the existence of a recently identified multipotent neutrophil-eosinophil cell type would imply that the existence of further neutrophil-eosinophil progenitors with novel phenotypes cannot be discounted and that the ability to differentiate into neutrophils or eosinophils may be much later in the differentiation pathway than is currently broadly accepted. Potentially, this unique neutrophil to eosinophil shift seen in wild mouse bone marrow may be indicative of wild cues driving these cell differentiations pathways that are not typically used by laboratory mice.

Comparing bone marrow neutrophils in laboratory and wild mice, CD11c provides a clear distinction (Fig. 3F–H), with CD11c+ proportions being higher in wild mice (*P* < 0.01). The functional role of this increased CD11c+ pool is unclear, however CD11c+ neutrophils have been observed during sepsis [39] and exhibit immunoregulatory properties [40]. CD11b expression by neutrophils in the wild mouse bone marrow, whilst not unanimously lower than all laboratory equivalents (*P* < 0.27), is extremely variable, ranging from 100% positive expression to 7% across the wild population. Potentially, the decrease in CD11b in wild mouse neutrophils could be an indication that many of these bone marrow neutrophils are immature (or neutrophil precursors), as has been observed in acute infection and during stress responses, wherein the proportion of immature neutrophils in the bone marrow dramatically increases due to mature neutrophil egress [41, 42].

### The range in bone marrow adiposity across laboratory mouse cohorts is equivalent to the range in wild mice

**Figure 4:**
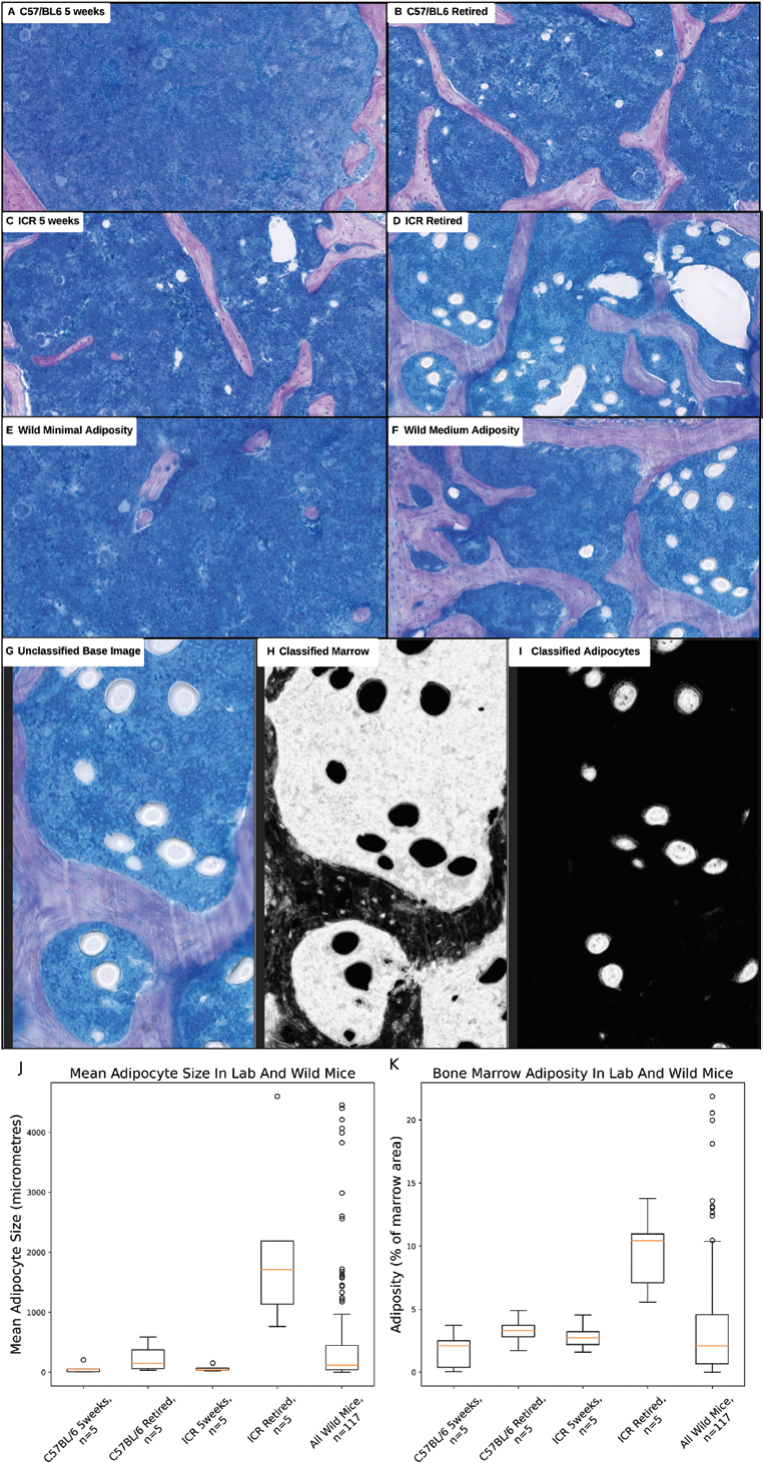
Analysis of adipocyte composition in laboratory and wild mouse bone marrow. Whole femurs from mice were decalcified, paraffin embedded, sectioned, and stained with Giemsa solution. All sections were cut from the central region of the femur (not proximal or distal). (A–G) Bone is stained purple. Marrow is stained blue (all nucleated cells). Adipocytes are spherical white (ghosts due to dehydration process). (G–I) Stained sections were imaged and assessed via WEKA segmentation to quantify marrow and adipocyte area. (G) Original image of a Giemsa-stained bone marrow sample from a wild mouse. (H) Classified image highlighting marrow exclusively in white. (I) Classified image highlighting adipocytes exclusively in white. (K) Adiposity was calculated by dividing the total area containing adipocytes by the total area containing marrow for each set of samples (one set per mouse), expressed as a percentage. (J and K) Box plots follow Tukey’s original definition: The orange line is the median, box limits show the interquartile range (IQR), whiskers are set to the closest value below 1.5 times the IQR, outliers (identified by being more than 1.5 times the IQR) are displayed as round circles. ‘5weeks’ mice are 5-week-old female mice, ‘retired mice’ are 6-month-old females with a history as stock breeders. ‘All IoM Mice’ includes all Isle of May wild mice for which data is present. *P*-values for each set of comparisons (each individual plot) were calculated by performing a Kruskal–Wallis test followed by a post-hoc Dunn’s test with Bonferroni correction and are included in Supplementary Table 2 (not displayed in figure).

It is well established that adiposity in the murine bone marrow increases with age [43] and that adipocytes have numerous roles in modulating haematopoiesis and immune function [44]. Consistent with these findings, retired (6 months old) laboratory mice had higher adiposity and larger adipocytes than their 5-week-old laboratory equivalents (Fig. 4J and K), though this is only statistically significant for some of the comparisons. Specifically, comparing C57BL/6 5 week old mice and C57BL/6 retired breeders shows a non-significant increase in adipocyte size (*P* = 0.16) and adiposity (*P* = 0.16). Comparing ICR 5 weeks and ICR retired breeders showed a significant increase in average adipocyte size (*P* < 0.01) but not adiposity (*P* = 0.07). A potential explanation for this inconclusive result is that this phenotype only arises in mice at a much older age of 1–2 years [16, 17].

Including the substantial number of outliers, the wild mouse population extends the full range of values found in the laboratory mouse groups for both adipocyte size and adiposity. As with the CD4+ T cell proportions, this indicates that the age ranges of the captured wild mice largely align with the 5 weeks to 6 months age ranges of the laboratory mice, which in turn aligns with the age estimates provided by Berry and Triggs *et al* [5–8] for the life expectancy of Isle of May mice.

### Only date of capture strongly delineates variation in immune variables in the wild mouse bone marrow

**Figure 5:**
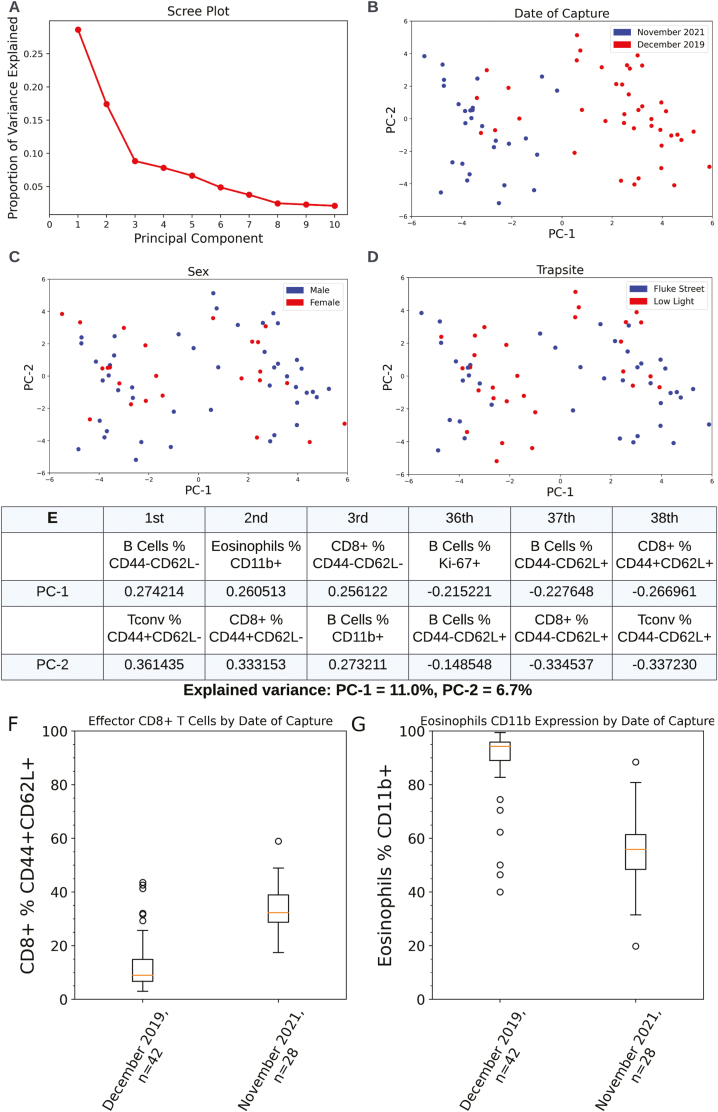
PCA plots of myeloid and lymphoid flow cytometry variables. Variables from myeloid panel B and lymphoid panel A (38 variables) were exported from Flowjo and used for PCA analysis. The sample set includes 70 mice total from November 2021 and December 2019. (A) Scree plot showing the proportion of variance explained by the first 10 principal components. (B–D) Principal component 1 is plotted on the x-axis, principal component 2 is plotted on the y-axis. Each sample (mouse) is represented as a dot. (B) Each sample is shaded by capture date. (C) Each sample is shaded by the sex of the mouse. (D) Each sample is shaded by capture site. (E) Table of eigenvalues for each of the principal components, only the top and bottom three are shown for brevity. (F–G) Boxplots of two major eigenvalue variables from principal component 1 from table 5E, by date of mouse capture. Box plots follow Tukey’s original definition: The orange line is the median, box limits show the interquartile range (IQR), whiskers are set to the closest value below 1.5 times the IQR, outliers (identified by being more than 1.5 times the IQR) are displayed as round circles. (F) *P* value as calculated by Mann Whitney U test between December 2019 and November 2021 is less than 0.01. (G) *P* value as calculated by Mann–Whitney U test between December 2019 and November 2021 is less than 0.01.

The bone marrow is well documented to be highly modulated by a wide variety of environmental and intrinsic variables, such as age [45], infection [46], or diet [47, 48], and wider literature (not bone marrow) provide a plethora of explanations for the appearance of novel immunological traits in a population. Both Brodin and Davis [49], in human populations, and Abolins *et al*. [50], in wild mice, agree that non-heritable variables are responsible for the majority of immune variation in a population (assessed from blood samples). To identify if there were key variables that could explain broad swathes of the variation in bone marrow immune phenotype across the wild mouse population, principal component analysis was run on flow cytometric variables from the myeloid and lymphoid compartments of the wild mouse bone marrow. The composition of the first two principal components and their alignment with date of capture, trap site, and sex are displayed in Fig. 5, also tested but not shown (due to being statistically insignificant) are blood packed cell volume, mouse body weight, mouse body length, liver weight, volume of subcutaneous fat, bone marrow adiposity, and *Trichuris* burden at cull.

The first key finding is that the only environmental/ecological variable to show delineation of the first two principal components is date of capture, specifically principal component 1 diverges between December 2019 and November 2021, whereas principal component 2 (Fig. 5B). While the composition of each principal component is complex (Fig. 5E), with each principal component representing the expression of 13 different biomarkers across six cell types (Supplementary Table 1), this is illustrative that the date of capture is broadly significant at determining overall immunological phenotype of the wild mouse bone marrow whilst other ecological/environmental variables are not. Figure 5F and G explore this further by plotting two of the major eigenvectors (flow cytometric variables) from principal component 1 against date of capture. Figure 5F illustrates a divergence in the proportion of effector CD44+CD62L+ CD8+ T cells between November 2021 and December 2019 (*P* < 0.01), whereas Figure 5G plots the percentage of CD11b+ eosinophils in the wild mouse bone marrow between capture dates, with a strong delineation between November 2021 and December 2019 (*P* < 0.01).

These two time points of November and December may reflect a crucial tipping point in ecological variables such as ambient temperature, food availability, infectious disease dynamics, and seasonal circadian rhythms, many of which are known to impact immune function [1–4, 8, 10, 14, 30, 46–50]. However, differences across year also need to be taken into consideration. Further delineating which of these variables are contributing to this broad division in bone marrow immunological phenotype will require further study of the ecological dynamics on the Isle of May and would greatly benefit from follow-up studies across the four seasons and across several years.

The second key finding relates to the extremely low explained variance provided by the first two principal components, 11.0% and 6.7%, respectively. This indicates that variation in the wild mouse bone marrow immune system is primarily characterized by small changes in large numbers of biomarkers and that there are no singular immunological variables that can be attributed to explaining the broad variation in immune phenotype. This multifaceted pattern of variation in the immune phenotype of the bone marrow is also further indication that the biological or ecological drivers of this variation are likely a dynamic collection of multiple variables.

## Conclusion

The wild mouse bone marrow differs from the laboratory mouse bone marrow in every major aspect measured; the proportions of mature leukocytes, proportions of leukocyte progenitors, proportion of adipocytes, and the phenotypes of mature leukocytes. Moreover, there are some features that have not been described before in a murine model. For example, the combination of Ly6C, Ly6G, and CD11c expression by eosinophils and neutrophils alongside a 5-fold increase in the eosinophil proportion in the bone marrow of some wild mice. This increased eosinophil population draws parallels with the work by Jeong *et al* [38]., indicating that further research into eosinophil/neutrophil progenitors is required, which may shed light on the dramatic swings in eosinophil to neutrophil ratios in the wild mouse bone marrow. However, while multiple aspects of the wild mouse bone marrow are novel to their laboratory mouse equivalents, what is of equal importance is that the wild mouse bone marrow is highly variable in phenotype across the population. Due to the large, diverse wild population, this article restricts its view to only the most significant findings. There are numerous less prominent phenotypes and unique wild mouse sub-populations that have not been displayed or discussed here. Further work investigating these novel sub-populations and uncovering mechanistic explanations for, and functional implications of these phenotypes is required.

This study has shown that the wider environment in which animals live has a major influence on bone marrow phenotype. Given the central role of the bone marrow within the immune system, ecological drivers of immune variation in this primary immune compartment are important to take into account when assessing the translatibility of existing laboratory models. We propose that future immunological research aims to bridge between mechanistic laboratory research in necessarily controlled settings, and holistic studies in more complex and natural environments. Doing so will improve the translatability, accountability, and the ultimate value of immunological research.

## Supplementary Material

kyad005_suppl_Supplementary_Data

kyad005_suppl_Supplementary_Figure_S1

kyad005_suppl_Supplementary_Figure_S2

kyad005_suppl_Supplementary_Figure_S3

kyad005_suppl_Supplementary_Figure_S4

kyad005_suppl_Supplementary_Table_S1

kyad005_suppl_Supplementary_Table_S2

kyad005_suppl_Supplementary_Table_S3

kyad005_suppl_Supplementary_Table_S4

## Data Availability

All data and extended methods pertaining to figures presented in this article is provided as Supplementary Data. Additional data is available upon request.

## Abbreviations:

CMP: common myeloid progenitor
FMO: fluorescence minus one
GMP: granulocyte-monocyte progenitor
HSC: haematopoietic stem cell
IFN: interferon
IL: interleukin
IoM: Isle of May
IQR: interquartile range
MEP: megakaryocyte–erythroid progenitor cell
NBF: neutral buffered formalin
PBS: phosphate buffered saline
SD: standard deviation
SEM: standard error of the mean

## Permissions to Reproduce

All text, figures and data presented in this article are free from external copyright. Complementary data arising from the same project have been published within a PhD thesis, due to be released as open access on 01/01/2024 by the University of Manchester titled ‘Characterising the wild mouse bone marrow: composition and cellular heterogeneity’.

## Clinical Trial Registration

The data and work presented in this article are not related to any currently registered clinical trial.
