# Supplementary material for: The wild mouse bone marrow has a unique myeloid and lymphoid composition and phenotype
Source: Discov Immunol. 2023 Apr 18;2(1):kyad005. doi: 10.1093/discim/kyad005 (PMC10917185; doi:10.1093/discim/kyad005)
Supplement: kyad005_suppl_Supplementary_Data [file kyad005_suppl_Supplementary_Data.docx]

**Supplementary Figure 1: Illustration of captured Isle of May mice; when, where and what was collected.**

Mice were captured across three different months and at two distinct and physically isolated trap sites (Fluke Street and Low Light). Two main types of samples pertaining to bone marrow were collected from each mouse, histology and flow cytometry. Up to three types of flow cytometry samples were collected, one pertaining to myeloid cells, one for lymphoid cells and one for haematopoietic progenitors. Note that this figure only illustrates data pertaining to bone marrow data collection and does not include what additional samples were collected from these mice that do not pertain directly to bone marrow research nor other IoM mice that may have been collected for other research purposes.

**Supplementary Figure 2: Gating strategy and antibodies used for flow cytometry panel A**

Illustration of the selection of marker expression used to define and phenotype the lymphoid compartment of the bone marrow for both laboratory and wild mice. First gate is at top-left. Sequence of gates is indicated by arrows and marked with text when multiple gates are present. Gates which define cell types have no coloured boundary, gates used to phenotype identified cell types are bordered by a coloured rectangle labelled with the name of the cell type being phenotyped. This figure uses examples from a single sample; however all samples utilised the same gating strategy. The defined Treg population (pink) was not phenotyped with any additional markers. Location of gates identifying positive versus negative staining were aligned using FMOs as negative controls (using FMOs prepared on the same day as the samples), gates identifying low, medium or high expression were aligned by observing the average expression across the entire dataset. All antibodies used for flow cytometry staining of panel A are listed: including, name of the conjugated fluorochrome, the manufacturers clone reference code and the name of correlating binding marker. All fluorochrome conjugated antibodies were purchased from Life Technologies.

**Supplementary Figure 3: Gating strategy and antibodies used for Myeloid panel B**

Illustration of the selection of marker expression used to define and phenotype the myeloid compartment of the bone marrow for both laboratory and wild mice. First gate is at top-left. Sequence of gates is indicated by arrows and marked with text when multiple gates are present. Gates which define cell types have no coloured boundary, gates used to phenotype identified cell types are bordered by a coloured rectangle labelled with the name of the cell type being phenotyped. This figure uses examples from a single sample; however all samples utilised the same gating strategy. DUMP includes the markers CD3, NKp46 and CD19. The defined Ly6C- macrophage (pink), Ly6C- monocyte (light blue) and Ly6C+ macrophage (purple) populations were phenotyped with additional markers but were not utilised within this project and therefore are not shown. Location of gates identifying positive versus negative staining were aligned using FMOs as negative controls (using FMOs prepared on the same day as the samples), gates identifying low, medium or high expression were aligned by observing the average expression across the entire dataset. All antibodies used for flow cytometry staining of panel B are listed: including, name of the conjugated fluorochrome, the manufacturers clone reference code and the name of correlating binding marker. All fluorochrome conjugated antibodies were purchased from Life Technologies.

**Supplementary Figure 4: Gating strategy and antibodies used for progenitor panel P**

Illustration of the selection of marker expression used to define and phenotype the myeloid compartment of the bone marrow for both laboratory and wild mice. First gate is at top-left. Sequence of gates is indicated by arrows and marked with text when multiple gates are present. Gates which define cell types have no coloured boundary, gates used to phenotype identified cell types are bordered by a coloured rectangle labelled with the name of the cell type being phenotyped. This figure uses examples from a single sample; however all samples utilised the same gating strategy. DUMP includes the markers CD3, NKp46 and CD19. The defined Ly6C- macrophage (pink), Ly6C- monocyte (light blue) and Ly6C+ macrophage (purple) populations were phenotyped with additional markers but were not utilised within this project and therefore are not shown. Location of gates identifying positive versus negative staining were aligned using FMOs as negative controls (using FMOs prepared on the same day as the samples), gates identifying low, medium or high expression were aligned by observing the average expression across the entire dataset. All antibodies used for flow cytometry staining of panel P are listed: including, name of the conjugated fluorochrome, the manufacturers clone reference code and the name of correlating binding marker. All fluorochrome conjugated antibodies were purchased from Life Technologies.

**Supplementary Table 1: Defining and phenotypic markers for the cell types used in this study.**

Defining markers are listed in order of gating. Most definitions include live/dead, forward scatter (size) and side scatter (density) parameters which are not included here but are illustrated in the gating strategies of supplementary figures 2, 3 and 4. Lin (lineage) includes the markers CD4, CD8, Ly6G, CD11b, TER-119, NK1.1, SiglecF and FcεRα1. Phenotypic markers are illustrated in figures 2 and 3.

**Supplementary Table 2: P values for main figures 1, 2, 3 and 4.**

p-values for each set of comparisons were calculated by performing a Kruskal-Wallis test followed by a post-hoc Dunn’s test with Bonferroni correction. Values are in order of appearance in the main figures. p<0.05 are shaded red, 0.05<p<0.01 are shaded yellow and p<0.01 are shaded green.

**Supplementary Table 3: Full dataset for main figures 1, 2, 3 and 4.**

Contains all data visualised in main figures 1, 2, 3 and 4.

**Supplementary Table 4: Demographics table for all wild mice.**

SMI (scaled mass index) was calculated using the same method as Peig & Green [1] and is a proxy for body condition. Age is estimated from dry eye lens weights following the method published by Rowe et al. [2]. Endoparasite infections were confirmed by visual confirmation of worms at cull. Faecal and liver samples were not available for all mice, hence the frequencies for each endoparasite infection do not add up to 124 (total number of mice analysed).

**References**

1. Peig J, Green AJ. New perspectives for estimating body condition from mass/length data: the scaled mass index as an alternative method. Oikos 2009, 118, 1883–91. doi:10.1111/j.1600-0706.2009.17643.x.

2. Rowe FP, Bradfield A, Quy RJ, Swinney T. Relationship between eye lens weight and age in the wild house mouse (Mus musculus). J Appl Ecol 1985, 55–61.
