# Supplementary figures and images for: The wild mouse bone marrow has a unique myeloid and lymphoid composition and phenotype
Source: Discov Immunol. 2023 Apr 18;2(1):kyad005. doi: 10.1093/discim/kyad005 (PMC10917185; doi:10.1093/discim/kyad005)

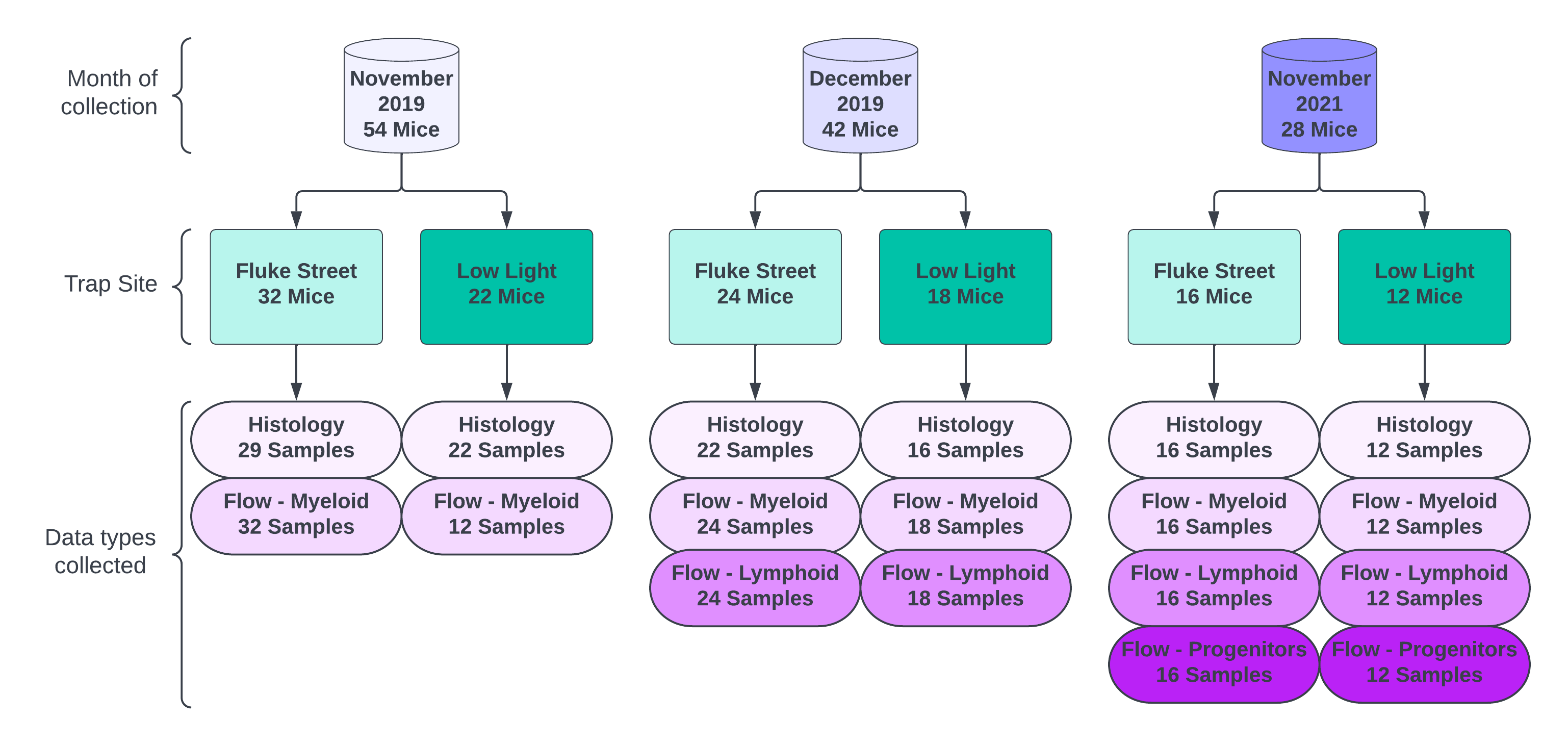

Supplement: kyad005_suppl_Supplementary_Figure_S1 [file kyad005_suppl_Supplementary_Figure_S1.png]

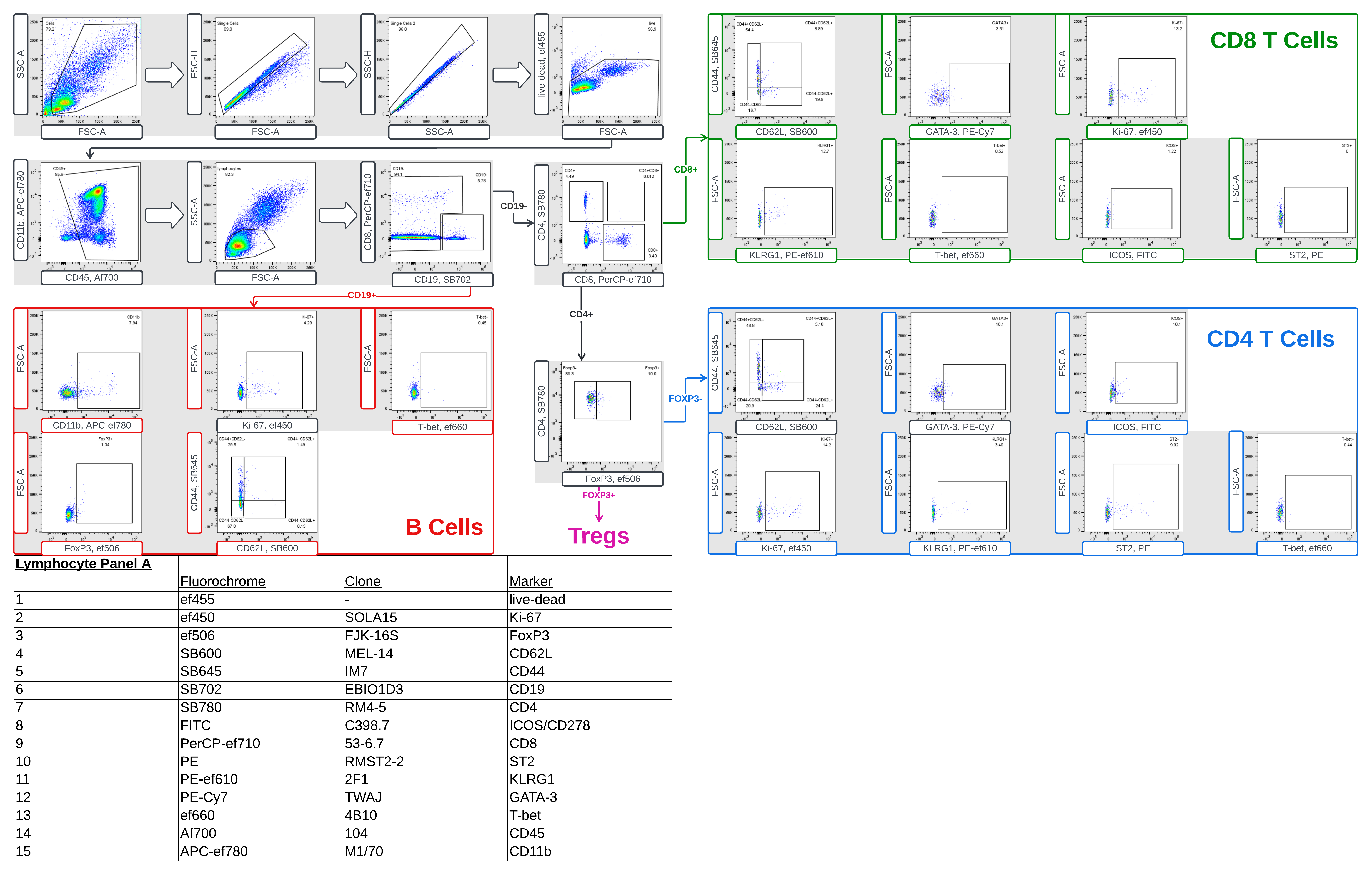

Supplement: kyad005_suppl_Supplementary_Figure_S2 [file kyad005_suppl_Supplementary_Figure_S2.png]

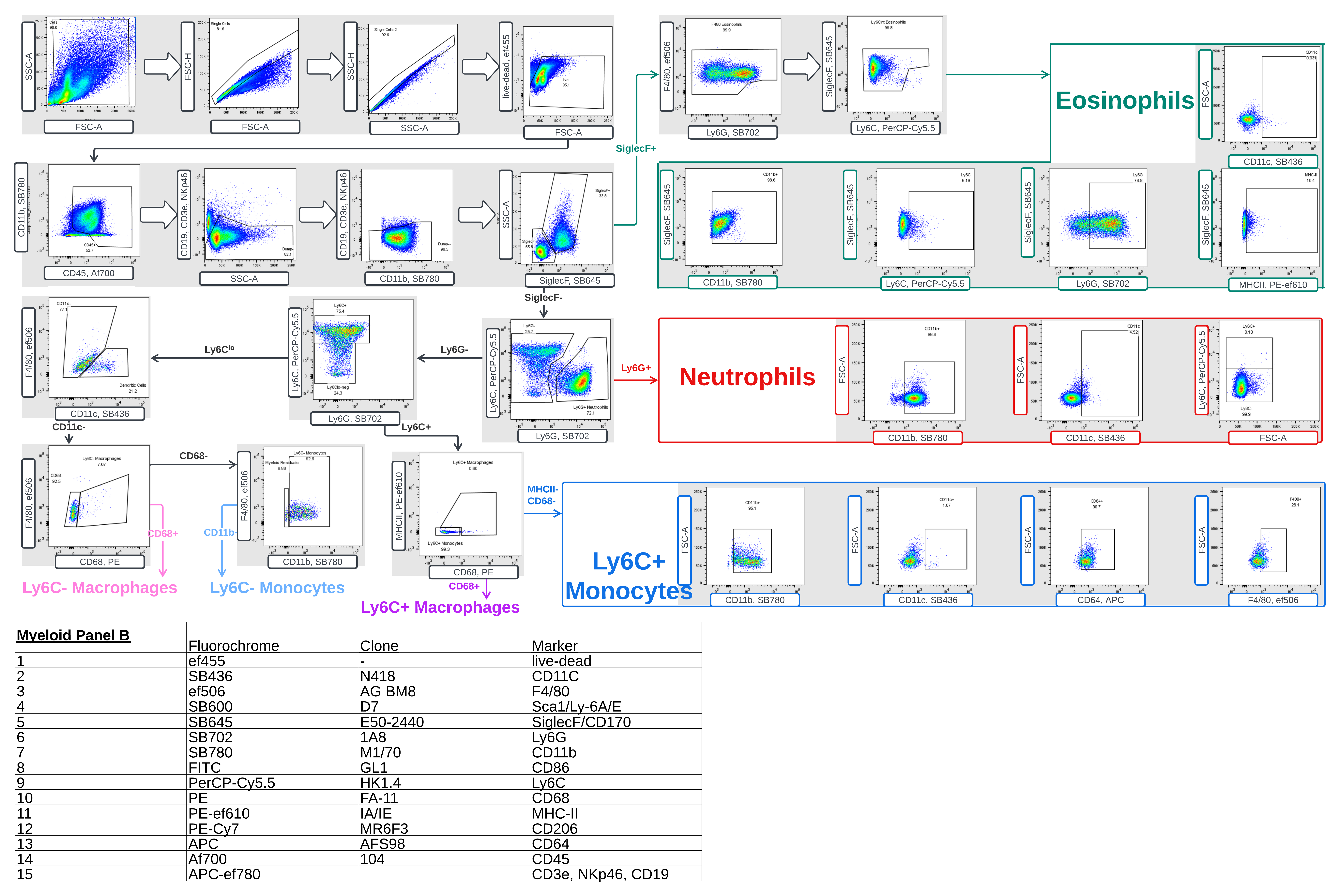

Supplement: kyad005_suppl_Supplementary_Figure_S3 [file kyad005_suppl_Supplementary_Figure_S3.png]

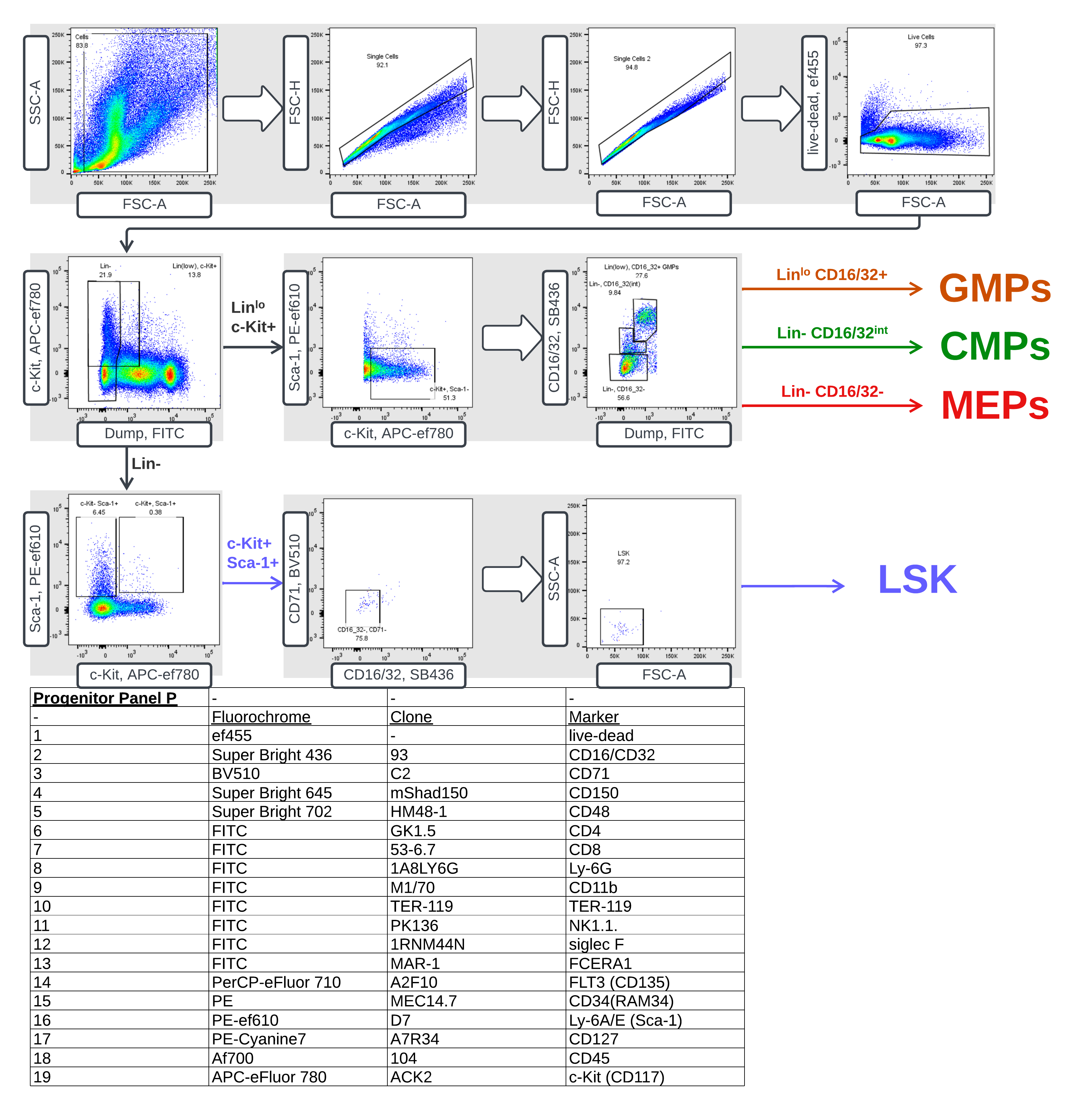

Supplement: kyad005_suppl_Supplementary_Figure_S4 [file kyad005_suppl_Supplementary_Figure_S4.png]
